# Hybrid Assistive Neuromuscular Dynamic Stimulation Therapy: A New Strategy for Improving Upper Extremity Function in Patients with Hemiparesis following Stroke

**DOI:** 10.1155/2017/2350137

**Published:** 2017-01-16

**Authors:** Toshiyuki Fujiwara, Michiyuki Kawakami, Kaoru Honaga, Michi Tochikura, Kaoru Abe

**Affiliations:** ^1^Department of Physical Medicine and Rehabilitation, Juntendo University Graduate School of Medicine, 2-1-1 Hongo, Bunkyo, Tokyo, Japan; ^2^Department of Rehabilitation Medicine, Tokai University School of Medicine, 143 Shimokasuya, Isehara, Kanagawa, Japan; ^3^Department of Rehabilitation Medicine, Keio University School of Medicine, 35 Shinanomachi, Shinjuku, Tokyo, Japan; ^4^Department of Rehabilitation Medicine, Keio University Hospital, 35 Shinanomachi, Shinjuku, Tokyo, Japan

## Abstract

Hybrid Assistive Neuromuscular Dynamic Stimulation (HANDS) therapy is one of the neurorehabilitation therapeutic approaches that facilitates the use of the paretic upper extremity (UE) in daily life by combining closed-loop electromyography- (EMG-) controlled neuromuscular electrical stimulation (NMES) with a wrist-hand splint. This closed-loop EMG-controlled NMES can change its stimulation intensity in direct proportion to the changes in voluntary generated EMG amplitudes recorded with surface electrodes placed on the target muscle. The stimulation was applied to the paretic finger extensors. Patients wore a wrist-hand splint and carried a portable stimulator in an arm holder for 8 hours during the daytime. The system was active for 8 hours, and patients were instructed to use their paretic hand as much as possible. HANDS therapy was conducted for 3 weeks. The patients were also instructed to practice bimanual activities in their daily lives. Paretic upper extremity motor function improved after 3 weeks of HANDS therapy. Functional improvement of upper extremity motor function and spasticity with HANDS therapy is based on the disinhibition of the affected hemisphere and modulation of reciprocal inhibition. HANDS therapy may offer a promising option for the management of the paretic UE in patients with stroke.

## 1. Functional Recovery of Upper Extremity Motor Function following Stroke

Stroke is a common health-care problem that causes physical impairment, disability, and problems in social participation. The most common impairment caused by stroke is motor impairment. Motor impairment affects the control of the unilateral upper and lower extremities. Recovery of function in the hemiparetic upper extremity is noted in fewer than 15% of patients after stroke [1].

Patients often compensate for their paretic upper extremity by using their intact upper extremity in the performance of everyday tasks [2]. It is supposed that strong reliance on compensatory overuse of the intact upper extremity inhibits functional recovery of the impaired upper extremity. This may explain the limited improvement of the functional capability of the paretic upper extremity in activities of daily living (ADL).

Principles of motor rehabilitation following stroke have been described as being dose-dependent and task-specific [3]. High-intensity practice and task-specific training are recommended for functional recovery. Several systematic reviews [4, 5] have explored whether high-intensity therapy improves recovery, and the principle that increased intensive training is helpful is widely accepted. Task-specific training is a well-accepted principle in motor rehabilitation. Training should target the goals that are relevant for the needs of the patients and preferably be given in the patient's own environment.

The goal of upper extremity rehabilitation is to improve the capability of the paretic upper extremity for ADL. Constraint-induced movement therapy (CIMT) has been developed to enhance the forced use of the paretic hand in ADL with reduction of the compensatory overuse of the intact upper extremity. However, to participate in CIMT, the candidates must be able to voluntary extend their fingers and wrist at least 10 degrees, practice for 6 hours daily in a 2-week course, and spend waking hours with their nonparetic hand in a mitt [6].

To counter potential problems inherent in the intensive services needed for CIMT, we developed an alternative therapeutic approach that provides high-intensity training to facilitate the use of the paretic upper extremity in daily living by combining closed-loop electromyography- (EMG-) controlled neuromuscular electrical stimulation (NMES) with a wrist-hand splint for patients with moderate to severe hemiparesis. Fujiwara et al. called this hybrid assistive neuromuscular dynamic stimulation (HANDS) therapy [7].

## 2. HANDS Therapy

A PubMed literature search was conducted using the MeSH terms stroke, rehabilitation, upper extremity function, and neuromuscular electrical stimulation, and 71 articles were identified. A further search of PubMed with the terms stroke, rehabilitation, upper extremity function, neuromuscular electrical stimulation, and splint identified 4 articles, all regarding HANDS therapy.

HANDS therapy facilitates the use of the paretic upper extremity in daily living by combining closed-loop EMG-controlled NMES with a wrist-hand splint for patients with moderate to severe hemiparesis. This HANDS system is active for 8 hours, and patients are instructed to use their paretic hand as much as possible while wearing the HANDS system. Their nonparetic upper extremity is not restrained. The patients are also instructed to practice bimanual activities in their ADL. All participants in HANDS therapy are admitted, and the length of the intervention is 21 days. They receive 90 minutes of occupational therapy per day, 5 days a week. Each session of occupational therapy consists of gentle stretching exercise of the paretic upper extremity and active muscle reeducation exercise. All participants are instructed how to use their paretic hand in ADL with the HANDS system. Occupational therapists are directed toward participants' goals and focused on their particular impairments and disabilities; thus, the specific therapy that each patient receives varies [7, 8].

Fujiwara et al. [7, 8] reported the indications for HANDS therapy as follows: (1) no cognitive deficits; (2) no pain in the paretic upper extremity; (3) passive extension range of motion (ROM) greater than 0 degrees of the affected wrist and −10 degrees of the metacarpophalangeal joints; (4) detectable surface EMG signals in the affected extensor digitorum communis (EDC) or extensor pollicis longus (EPL) when the patient intends to extend their fingers; (5) ability to raise the paretic hand to the height of the nipple; (6) scores of Fugl-Meyer test position sense of joints in the glenohumeral joint, elbow, wrist, and thumb of 1 or more; and (7) the ability to walk without physical assistance in daily life (e.g., including patients who can walk independently with a cane and/or an orthosis). The exclusion criteria were (1) history of major psychiatric or previous neurological disease, including seizures; (2) cognitive impairment precluding appropriately giving informed consent or the patient's Mini Mental Examination Scale score was below 25; (3) patients with severe pain in the paretic upper extremity; (4) patients with a pacemaker or other implanted stimulator; and (5) patients with visuospatial neglect or apraxia.

Previous reports showed that none of the patients experienced any discomfort or significant disability with the HANDS therapy.

### 2.1. Closed-Loop Electromyography- (EMG-) Controlled Neuromuscular Electrical Stimulation (NMES)

Twenty-nine articles were found in PubMed using the terms stroke, electromyography, neuromuscular electrical stimulation, and upper extremity. Thirteen of 29 articles were on EMG-triggered NMES. Six of 29 articles were on EMG-controlled NMES. Two involved contralaterally controlled electrical stimulation.

EMG-triggered NMES applies preset electrical stimulation when EMG activity reaches a target threshold. The stimulus intensity and duration are determined and not changeable. EMG-controlled NMES applies electrical stimulation during voluntary contraction and changes the stimulation intensity in proportion to the changes in EMG amplitude.

**Figure 1:**
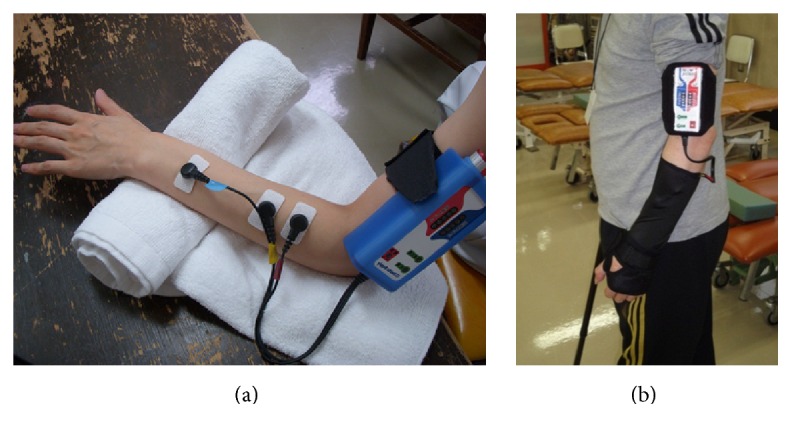
(a) Closed-loop electromyography- (EMG-) controlled neuromuscular electrical stimulation (NMES). A pair of electrodes for EMG detection and stimulation are placed on the affected extensor digitorum communis muscle, and one electrode for stimulation is placed on the affected extensor indicis muscle. (b) Participants wear a wrist-hand splint and carry a closed-loop EMG-controlled NMES with arm holder for 8 hours during the daytime.

For assistive stimulation, HANDS therapy used closed-loop EMG-controlled NMES, which was developed by Muraoka [9] and commercially available with MURO stimulation (Pacific Supply, Osaka, Japan). This closed-loop EMG-controlled NMES is portable and attaches to the arm (Figure 1). The surface electrodes pick up EMG signals at the target muscle and simultaneously stimulate it in direct proportion to the picked-up EMG signal, with the exception of the 25 ms after delivering each stimulation pulse, in which stimulation artifacts and M wave are observed. The external adjustment unit sets (1) range of stimulus intensity; (2) sensitivity of the EMG; (3) threshold of EMG amplitude that starts stimulation; and (4) gradient of stimulus intensity change to the change of EMG amplitude. Once these parameters were set with the external adjustment unit, the stimulator memorized these parameters.

It is difficult for patients with severe to moderate hemiparesis to extend their paretic fingers. As for hand function to perform ADL, pinch and release, and grip and release, are key functions. It is necessary to restore finger extension to perform ADL with the paretic upper extremity in patients with severe to moderate hemiparesis. To restore finger extension, electrical stimulation is applied to finger extensors in HANDS therapy. A pair of electrodes for EMG detection and stimulation (10 mm diameter) placed 20 mm apart on the affected EDC and one electrode (10 mm) for stimulation are placed on the affected EI.

The EMG data and amount of stimulation were recorded with an attached data-logger system of the MURO device while the participants wore the MURO device. The participant's compliance with wearing the device for 8 hours during the daytime can be monitored using this data-logger system in HANDS therapy.

### 2.2. Splint

The patients wear a wrist-hand splint (Wrist Support, Pacific Supply Co.) and carry a portable closed-loop EMG-controlled NMES with arm holder for 8 hours during the daytime. The rationale for combining the stimulation system with a wrist-hand splint was derived from the work of Fujiwara et al. [10]. They showed that wearing a wrist-hand splint reduced spinal motoneuron excitability and flexor muscle overactivity during voluntary finger extension. During finger extension, muscle activities of the finger flexors, wrist flexors, and elbow flexors were reduced with the wrist-hand splint. The wrist-hand splint effect on the elbow flexors was supposed to be mediated by secondary afferent inhibition [11].

The wrist-hand splint also makes the hand shape functional. Hand shape is important for hand function. The hand has longitudinal and transverse arches. These arches are important for holding, and thumb opposition and the web space are important for pinching. A wrist-hand splint helps to form the longitudinal and transverse arches, thumb opposition, and the web space in the hand [10].

## 3. The Effect of HANDS Therapy

Shindo et al. [12] performed a randomized, controlled study among subacute patients (time from stroke onset within 60 days) with hemiparesis following stroke. They explored the effectiveness of HANDS therapy added to conventional rehabilitation as compared with splint therapy in addition to standard inpatient rehabilitation treatment for patients who could not fully extend their paretic fingers and could not perform pinch and release in their daily life, in a randomized, controlled trial design. Compared with the control group, the HANDS group showed significantly greater gains in the distal (hand/wrist) part of the Fugl-Myer Assessment (FMA) [13] and improvement of the Action Research Arm Test [14]. HANDS therapy is an intervention that resulted in improved hand function following stroke, while a systematic review [3] showed that none of the interventions identified showed a consistent pattern of improvement in hand function.

HANDS therapy improved upper extremity function even in patients with chronic stroke [7, 8]. Fujwara et al. [8] applied HANDS therapy to 61 patients with chronic hemiparetic stroke. Their mean time since stroke onset was 28.4 months. Three weeks of HANDS therapy improved FMA, the motor activity log 14 (MAL) amount of use score [15], and the modified Ashworth scale (MAS) [16]. Improvement of the FMA, MAL, and MAS lasted for 3 months after the end of HANDS therapy. In the study of Fujiwara et al. [7], arm and finger functions were assessed with the Stroke Impairment Assessment Set (SIAS) motor function score [17]. They found that both arm and hand function had been improved by HANDS therapy, and these improvements were maintained until 3 months after the end of HANDS therapy. They also showed improved capability of the paretic hand in ADL.

These studies showed that HANDS therapy improved arm and hand motor functions, increased the amount of use of the paretic upper extremity in ADL, and reduced finger and wrist spasticity, not only in subacute, but also in chronic stroke. The mean FMA gains with HANDS therapy were 12.2 in subacute patients [12] and 7.7 in chronic patients [8]. These gains surpassed the minimal clinically important difference for treatment-induced gains of 4.25 on the FMA [18].

## 4. The Mechanism of Functional Recovery and Neural Plasticity Induced with HANDS Therapy

Dose-dependent, task-specific, and use-dependent plasticity are principles of rehabilitation for functional recovery.

HANDS therapy improved motor function and increased the amount of paretic hand use. These improvements were maintained until 3 months after the end of HANDS therapy. These long-lasting effects of HANDS therapy can be explained by the concept of the threshold of effective rehabilitation, which was proposed by Han et al. [19]. If spontaneous arm use is above a certain threshold, then training can be stopped, as repeated spontaneous use provides a form of motor learning that further improves performance and spontaneous use. Below this threshold, training is in vain, and compensatory movements with the less affected hand are reinforced. In HANDS therapy, participants were trained to use their paretic hand for 8 hours in 3 weeks using closed-loop EMG-controlled NMES and a hand splint. Such an amount of training may be above the threshold of effective rehabilitation.

The effect of training is task-specific [20]. The aim of HANDS therapy is to make the paretic hand useful for ADL and to have the paretic hand participate in ADL. The key functions of the hand in ADL are grip and release and pinch and release. It is difficult for patients with moderate or severe hemiparesis to extend their fingers. The closed-loop EMG-controlled NMES, therefore, helps to extend the paretic fingers, and the splint helps the patient pinch and hold the objects with paretic fingers. Using this HANDS system, participants were trained to use their paretic hand in their ADL, producing proximal and distal coordinated movements, such as reach, grip and release, and pinch and release.

One of the mechanisms of functional recovery of stroke is use-dependent plasticity. Functional recovery involves changes in neuronal excitability that alter the brain's representation of motor and sensory functions. The inhibitory neurotransmitter GABA is critical for cortical plasticity. In animal studies, reducing GABA_A_ergic inhibition proved beneficial for functional recovery [21, 22]. In humans, this GABA_A_ergic inhibitory system can be assessed with a paired-pulse transcranial magnetic stimulation (TMS) technique, in which a conditioning TMS pulse below the threshold for eliciting a motor-evoked potential (MEP) inhibits a suprathreshold test stimulus at short intervals (1–5 ms) (short intracortical inhibition (SICI)) [23]. Fujiwara et al. [8] showed that HANDS therapy induced disinhibition of SICI in the affected hemisphere, and there was a direct correlation between the change of SICI in the affected hemisphere and the change of FMA. Patients who showed more disinhibition of SICI showed longer lasting improvement of motor impairment. It has been supposed that long-lasting functional reorganization of the brain may be mediated by disinhibition of intracortical inhibitory interneurons in severely hemiparetic patients. In moderate to severe hemiparesis, compensatory brain responses include increased activation in the surrounding damaged zone and masked network [24]. HANDS therapy strengthened disinhibition of the affected SICI. It is thought that compensatory disinhibition occurred in moderate to severe chronic stroke during functional recovery induced with HANDS therapy. The mechanism of functional recovery of the upper extremity is not able to be explained by the disinhibition of SICI alone. More is necessary to induce functional recovery.

HANDS therapy improved the spasticity of the fingers and wrist. Spasticity is often blamed for poor function in patients with minimal finger extension but some preservation of flexion [20]. The mechanisms underlying spasticity poststroke have not been fully elucidated, but decreased reciprocal inhibition may contribute to motor impairment in spastic hemiparesis [7]. In healthy subjects, group Ia-mediated reciprocal inhibition contributes to the suppression of antagonist muscle activity during movement [25, 26]. This reciprocal inhibition is disrupted among patients with spastic hemiparesis [7, 27]. HANDS therapy reduced cocontraction of finger flexors during finger extension movement. This may be due to the restoration of reciprocal inhibition with HANDS therapy. Fujiwara et al. [8] studied reciprocal inhibition with the flexor carpi radialis H reflex conditioning-test paradigm [28] before and after HANDS therapy. HANDS therapy increased the magnitude of presynaptic inhibition and long loop presynaptic inhibition. They found a significant correlation between restoration of RI and improvement of wrist spasticity.

Disinhibition of affected intracortical interneurons increases the activity of the descending projection from the affected hemisphere to the spinal cord. Increased activities of the descending projection to the spinal cord modulate the activities of reciprocal inhibitory interneurons [29, 30].

More evidence is needed to investigate the neural plasticity changes underlying functional improvement after HANDS therapy by using brain imaging techniques such as fMRI.

HANDS therapy was applied in subacute and chronic stroke patients. There was no report of adverse effects. We consider that HANDS therapy is suitable for subacute and chronic phase of stroke and patients with synergy level, who cannot extend their paretic finger enough to use their paretic hand in their ADL.

## 5. Conclusion

HANDS therapy is one of the neurorehabilitation therapeutic approaches that facilitates the use of the paretic upper extremity in daily life by combining closed-loop EMG-controlled NMES with a wrist splint. Functional recovery from stroke has been induced with HANDS therapy even in chronic and moderate to severe hemiparesis. The improvements of motor function and spasticity induced by HANDS therapy are based on cortical and spinal plastic changes.

As the other NMES, HANDS therapy may offer a promising option for the management of the paretic upper extremity in patients with stroke. Further development and clinical application of HANDS therapy are needed.
